# Mechanical stretch up-regulates the B-type natriuretic peptide system in human cardiac fibroblasts: a possible defense against transforming growth factor-β mediated fibrosis

**DOI:** 10.1186/1755-1536-5-9

**Published:** 2012-07-07

**Authors:** Chris J Watson, Dermot Phelan, Maojia Xu, Patrick Collier, Roisin Neary, Albert Smolenski, Mark Ledwidge, Kenneth McDonald, John Baugh

**Affiliations:** 1 School of Medicine & Medical Science, The Conway Institute of Biomolecular and Biomedical Research, University College Dublin, Dublin, Ireland; 2 Heart Failure Unit, St Vincent’s University Hospital Healthcare Group, Elm Park, Dublin, Ireland

**Keywords:** Mechanical stretch, BNP, Natriuretic peptide receptor A, Transforming growth factor beta, Myofibroblast, Alpha smooth muscle actin

## Abstract

**Background:**

Mechanical overload of the heart is associated with excessive deposition of extracellular matrix proteins and the development of cardiac fibrosis. This can result in reduced ventricular compliance, diastolic dysfunction, and heart failure. Extracellular matrix synthesis is regulated primarily by cardiac fibroblasts, more specifically, the active myofibroblast. The influence of mechanical stretch on human cardiac fibroblasts’ response to pro-fibrotic stimuli, such as transforming growth factor beta (TGFβ), is unknown as is the impact of stretch on B-type natriuretic peptide (BNP) and natriuretic peptide receptor A (NPRA) expression. BNP, acting via NPRA, has been shown to play a role in modulation of cardiac fibrosis.

**Methods and results:**

The effect of cyclical mechanical stretch on TGFβ induction of myofibroblast differentiation in primary human cardiac fibroblasts and whether differences in response to stretch were associated with changes in the natriuretic peptide system were investigated. Cyclical mechanical stretch attenuated the effectiveness of TGFβ in inducing myofibroblast differentiation. This finding was associated with a novel observation that mechanical stretch can increase BNP and NPRA expression in human cardiac fibroblasts, which could have important implications in modulating myocardial fibrosis. Exogenous BNP treatment further reduced the potency of TGFβ on mechanically stretched fibroblasts.

**Conclusion:**

We postulate that stretch induced up-regulation of the natriuretic peptide system may contribute to the observed reduction in myofibroblast differentiation.

## Background

Hypertensive heart disease describes a phase of remodeling which occurs in the myocardium when exposed to sustained elevation in arterial blood pressure or hypertrophic substances associated with the hypertension syndrome. The two most notable features of hypertensive heart disease are myocyte hypertrophy and reactive fibrosis. The relationship between reactive fibrosis and hypertension is well described [1-3].

In physiological terms, the effect of this fibrosis is a reduction in compliance of the myocardium and diastolic dysfunction. This premise is based on two principles. Firstly, the addition of fibrillar collagen to normal tissue results in reduced compliance of that tissue, and secondly, regression of such fibrosis improves compliance and reduces cardiac stiffness [4-8]. The synthesis and degradation of fibrillar collagen is regulated by the myocardial fibroblast. Fibroblast differentiation to the more active myofibroblast form is a hallmark of cardiac fibrosis, and is associated with increased collagen production, enhanced proliferative and migratory potential, and is associated with increased expression of alpha-smooth muscle actin (ASMA) [9]. Although myofibroblast differentiation is an essential process required for normal wound healing, prolonged injury or perhaps loss of regulation can result in pathological fibrosis.

Modulation of pro-fibrotic signals within the myocardium is necessary to regulate normal wound healing processes. One possible regulator may be B-type natriuretic peptide (BNP). BNP is an endogenous hormone which is known to be secreted by cardiac myocytes in response to myocardial stretch and overload and is an important regulator of blood volume homeostasis through its diuretic, natriuretic, and vasodilating actions and by inhibiting renin and aldosterone [10,11]. More recent data suggest that natriuretic peptides also appear to play a major role in modulation of both cardiac and renal fibrosis [12-14].

The aim of this study was to investigate the combined effects of mechanical stretch and the pro-fibrotic cytokine transforming growth factor beta (TGFβ) on myofibroblast differentiation, and the impact of BNP in this process.

## Results

The impact of mechanical stretch on human primary cardiac fibroblast cells response to recombinant TGFβ was investigated. These experiments were carried out on flexible six well culture plates coated with 2 μg/cm^2^ human fibronectin, as described in the Methods section. Cells at a confluency of approximately 70% were exposed to cyclic equibiaxial Heart Simulation strain (1 Hz, maximum elongation 10%) for 72 h. Control plates were exposed to the same conditions without being stretched. These experiments were carried out either in the presence or absence of 10 ng/mL recombinant TGFβ.

### TGFβ-induced ASMA and collagen 1 is reduced on mechanically stretched cardiac fibroblast cells

**Figure 1:**
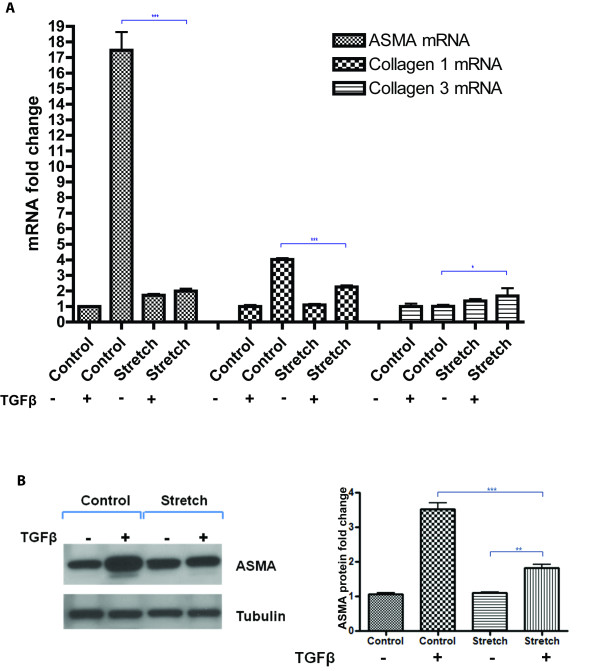
**Mechanical stretch reduces TGFβ induction of alpha-smooth muscle actin (ASMA) and collagen 1 expression in human cardiac fibroblasts.** Human primary cardiac fibroblasts underwent either biaxial mechanical stretch for 72 h or were left un-stretched (control), in the presence of 10 ng/mL transforming growth factor beta (TGFβ). Alterations in ASMA, collagen 1, and collagen 3 gene expression were examined using quantitative real-time PCR analysis (**A**). Western blotting was used to quantify changes in ASMA protein expression (**B**). Anti-tubulin was utilized as a loading control for western blotting, allowing for semi-quantitative densitometry analysis. Bar graphs represent mean fold change ± SD. *P < 0.05, **P < 0.01, ***P < 0.001.

The combined effects of mechanical stretch and TGFβ treatment on human primary cardiac fibroblast expression of the differentiation marker ASMA and the pro-fibrotic markers collagen 1 and 3 were investigated. Quantitative real-time PCR analysis revealed that TGFβ induction of ASMA gene expression was significantly reduced on stretched cells. TGFβ treatment for 72 h on non-stretched cells up-regulated ASMA by over 15-fold, whereas TGFβ stimulated ASMA expression was only two-fold in stretched cells, *P* < 0.001 (Figure 1A). A similar response was detected for collagen 1, where TGFβ induction of collagen 1 gene expression was reduced from approximately four-fold to two-fold when cells were mechanically stretched compared to non-stretched cells, *P* < 0.001 (Figure 1A). Interestingly, unlike ASMA and collagen1, TGFβ induction of collagen 3 expression was significantly enhanced when cells were mechanically stretched, *P* < 0.05. Due to the magnitude of the suppressive effects of stretch on TGFβ up-regulation of ASMA, this observation was verified at the protein level by Western blotting, where TGFβ induced ASMA was significantly diminished in stretched cells, *P* < 0.001 (Figure 1B).

### Diminished responses to exogenous TGFβ on mechanially stretched cardiac fibroblast cells are not due to decreased basal expression levels of TGFβ

**Figure 2:**
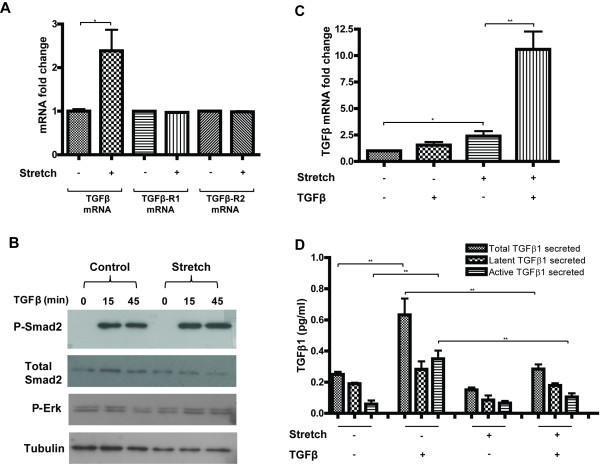
**Mechanical stretch does not reduce basal TGFβ expression in human cardiac fibroblast cells.** Human primary cardiac fibroblast cells underwent either biaxial mechanical stretch for 72 h or were left un-stretched (control), in the presence or absence of 10 ng/mL transforming growth factor beta (TGFβ). Analysis of stretch induced basal gene expression changes of TGFβ, TGFβ receptor type-1 (TGFβ-R1), and TGFβ receptor type-2 (TGFβ-R2) by quantitative real-time PCR are highlighted (**A**). Presented gene expression fold changes in Figure A are independent of recombinant TGFβ treatment and represent the impact of mechanical stretch alone. Western blot analysis of the impact of 72 h of mechanical stretch on TGFβ induced phosphorylation of Smad2 and ERK protein expression (**B**). Quantitative real-time PCR analysis of the effect of exogenous TGFβ treatment on basal TGFβ expression (**C**). The impact of mechanical stretch and recombinant TGFβ treatment on cardiac fibroblast production and secretion of total and active TGFβ protein was assessed using ELISA-based methods (**D**). Bar graphs represent mean fold change ± SD. *P < 0.05, **P < 0.01.

The impact of cyclical mechanical stretch on basal and TGFβ stimulated expression of TGFβ, and its receptor subtypes 1 and 2 (TGFβ-R1; TGFβ-R2) were investigated. Human cardiac fibroblast cells underwent mechanical stretch for 72 h in the presence or absence of 10 ng/mL TGFβ. Quantitative gene expression analysis revealed that mechanical stretch did not alter the levels of TGFβ receptors (Figure 2A). However, mechanical stretch did impact the basal expression levels of TGFβ. A significant 2.5-fold increase in gene expression was observed (*P* < 0.05) (Figure 2A). Further evidence that the diminished response to exogenous TGFβ on mechanically stretched cells was not due to reduced basal expression of TGFβ, or its receptors, is shown in Figure 2B. A reduction in Smad2 phosphorylation was not observed under stretched conditions. Of note, treatment with TGFβ in stretched cells resulted in a 10-fold increase in basal expression of TGFβ gene expression (*P* < 0.01), Figure 2C, but had no impact on expression of its receptors (data not shown). The impact of mechanical stretch and recombinant TGFβ treatment on HCF production of TGFβ protein production was investigated. Both total and active TGFβ1 was quantified in the supernatant of HCF using ELISA based methods (Figure 2D). The relatively large increase in TGFβ mRNA expression in cells exposed to mechanical stretch and recombinant TGFβ stimulation was not reflected at the protein level. The highest concentration of secreted TGFβ protein (both latent and active) was in un-stretched cardiac fibroblast cells stimulated with recombinant TGFβ treatment for 72 h. The impact of stretch alone did not have a significant impact on TGFβ secretion (although a downward trend was apparent). However, the ability of recombinant TGFβ to stimulate endogenous TGFβ production was reduced when the cells were mechanically stretched. Interestingly however, it is important to note that the detectable levels of secreted TGFβ were <1 pg/mL, thus the physiological relevance of these small changes within the experimental cell culture environment is unknown, especially under conditions of recombinant TGFβ treatment were cells are exposed to 10,000x this concentration.

Collectively, these data suggest that the observed reduction in TGFβ-mediated myofibroblast differentiation on mechanically stretched cells does not occur through a reduction in gene expression levels of TGFβ or receptor subtype 1 or 2. Concentration differences in secreted TGFβ are minimal and are unlikely to be responsible for the observed reduction of TGFβ induction of ASMA in stretched cells highlighted in Figure 1. This is further supported by intact Smad2 and ERK phosphorylation in response to short time point stimulation with TGFβ (15 and 45 min) post 72 h mechanical stretch.

### Mechanical stretch up-regulates both BNP and its receptor NPRA in primary human cardiac fibroblast cells

**Figure 3:**
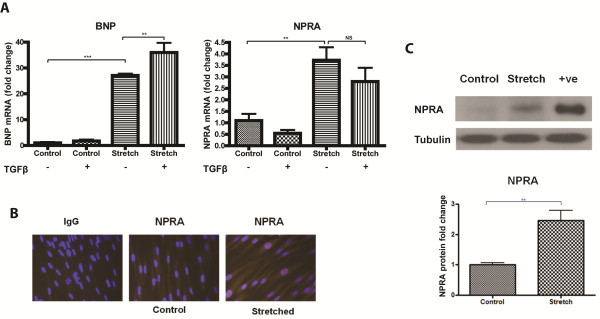
**Mechanical stretch up-regulates BNP and natriuretic peptide receptor A (NPRA) in human cardiac fibroblasts.** Human primary cardiac fibroblast cells underwent either biaxial mechanical stretch for 72 h or were left un-stretched (control) in the presence or absence of 10 ng/mL recombinant TGFβ. Changes in gene expression of b-type natriuretic peptide (BNP) and its receptor NPRA were investigated using quantitative real-time PCR (**A**). Further evidence that mechanical stretch has the ability to up-regulate NPRA is demonstrated at the protein level in the absence of TGFβ stimulation. Increased NPRA protein on stretched cardiac fibroblasts is evident, as shown by immunofluorescent microscopy (**B**). The intensity of NPRA immunofluorescence (red) is greater in cells mechanically stretched for 72 h compared to un-stretched (control) cells. The nuclei of cells were counterstained using DAPI (blue). Images were captured (Zeiss Axio Imager M1), at the same exposure time for comparison. Stretch-induced (72 h) up-regulation of NPRA protein was measured by western blotting and quantified using densitometry (**C**). Protein extracted from HEK-293-T cells over-expressing NPRA were used as a positive control (+ve). Bar graphs represent mean fold change ± SD. **P < 0.01, ***P < 0.001.

Cardiac fibroblast cells exposed to mechanical stretch for 72 h exhibited a significant increase in gene expression of BNP and its receptor natriuretic peptide receptor A (NPRA). BNP and NPRA were significantly up-regulated by over 25-fold (*P* < 0.001) and almost four-fold (*P* < 0.01), respectively (Figure 3A). TGFβ treatment in non-stretched conditions did not significantly impact BNP or NPRA gene expression levels. Interestingly, TGFβ treatment in the presence of stretch further increased the expression level of BNP. A trend towards a negative impact of TGFβ treatment on NPRA gene expression was observed. NPRA protein expression was apparent in both non-stretched and stretched fibroblast cells by immunofluorescence microscopy. However, the intensity of positive immuno-staining was greater in cells that underwent mechanical stretch (Figure 3B). Quantitative evidence of increased NPRA protein expression in stretched cardiac fibroblasts was observed using western blotting. NPRA protein expression was up-regulated more than two-fold following 72 h of mechanical stretching (*P* < 0.01) (Figure 3C). Cell lysates derived from transient over-expression of NPRA served as a positive control.

### Exogenous BNP further reduces the pro-fibrotic effects of TGFβ on stretched primary human cardiac fibroblast cells

**Figure 4:**
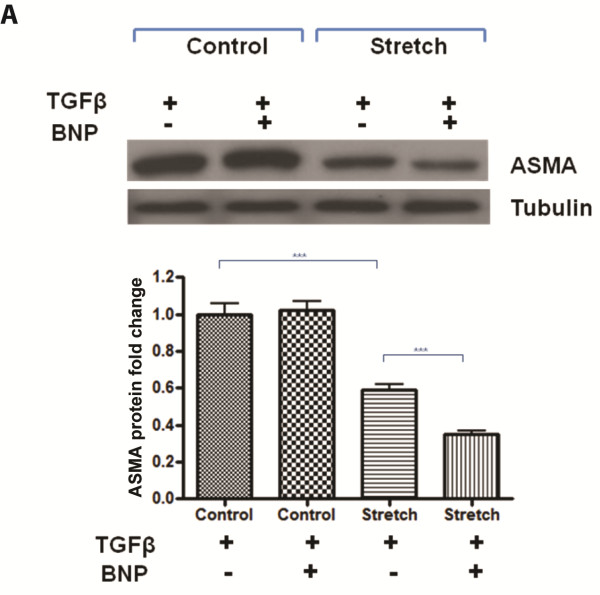
**Treatment with exogenous BNP further reduces the pro-fibrotic effects of TGFβ on mechanically stretched fibroblasts.** Human primary cardiac fibroblast cells underwent either biaxial mechanical stretch for 72 h or were left un-stretched (control). During this time, cells were either exposed to 10 ng/mL transforming growth factor beta (TGFβ) or 1,000 nM recombinant b-type natriuretic peptide (BNP), or both. Changes in alpha-smooth muscle actin (ASMA) protein expression were measured using western blotting and quantified using densitometry. Anti-tubulin was utilized as a loading control for western blotting. Bar graphs represent mean fold change ± SD. ***P < 0.001.

The reduced potency of TGFβ to induce myofibroblast differentiation on stretched cardiac fibroblasts was further investigated in the context of stretch induced BNP and NPRA expression. The impact of recombinant BNP treatment in combination with TGFβ on stretched cells was explored. It was found that the anti-fibrotic effects of exogenous BNP, as measured by ASMA protein repression, were only apparent when cardiac fibroblasts were mechanically stretched for 72 h (Figure 4).

## Discussion

TGFβ is a powerful mediator of adverse cardiac remodeling and fibrosis primarily via Smad-dependent induction of collagen expression as well as differentiation of fibroblasts to the myofibroblast phenotype which exhibits increased secretory, migratory, and proliferative properties and express the contractile protein ASMA [15-18]. The current study presents a novel observation that the pro-fibrotic effects of TGFβ are reduced on mechanically stretched human primary cardiac fibroblasts. In addition to this, we also document for the first time that mechanical stretch can up-regulate BNP and NPRA expression in human primary cardiac fibroblast cells, which could have important implications in modulating myocardial fibrosis. This point is emphasized by the observed increased anti-fibrotic effect of exogenous BNP treatment on stretched fibroblasts.

Within a normal or exaggerated wound healing environment additional stimuli are present along with pro-fibrotic factors such as TGFβ which may impact cellular activities. These may include the impact of tissue ischemia, circulating neuro-hormonal factors, or mechanical stretch on cellular behavior. A pressure or volume overloaded heart may exert increased mechanical strain at a cellular level. In combination with a pro-fibrotic environment, these additional heart stresses may impact disease progression, such as cardiac fibrosis and tissue remodeling.

One component of this study was to determine whether the impact of TGFβ on primary human cardiac fibroblasts was different on mechanically stretched cells compared with static cells. Surprisingly, we found that TGFβ induced ASMA expression was reduced when cells underwent cyclical stretch compared to static cells. This was also apparent for collagen 1 gene expression, but not collagen 3. Based on previous work using various cardiovascular cell types from a range of animal species, we anticipated that the combination of mechanical stretch and TGFβ treatment would have increased ASMA and collagen 1 expression compared with TGFβ treated static cells resulting in an enhanced pro-fibrotic effect [19-21]. Interestingly, our finding that mechanical stretch reduces myofibroblast differentiation has recently been supported by similar observations in primary lung fibroblasts [22]. Here, Blaauboer et al. investigated the impact of sinusoidal stretch on lung fibroblast response to TGFβ. The authors suggest that their observed reduction in the ability of exogenous TGFβ to induce ASMA was due to a stretch mediated down-regulation of basal TGFβ gene expression. However, we do not observe this in our system with primary human cardiac fibroblast cells. In fact, we observe an increase in basal levels of TGFβ gene expression. However, when examining the protein levels of TGFβ produced and released by the HCF, it was observed that the ability of recombinant TGFβ to stimulate endogenous TGFβ production was reduced when the cells were mechanically stretched. It is important to note that the detectable levels of secreted TGFβ were <1 pg/mL, which likely rules out a paracrine effect of TGFβ released from the HCF being the driver of the reduced myofibroblast differentiation in the presence of stretch and recombinant TGFβ. Within an *in vivo* setting the main source of TGFβ driving myofibroblast differentiation in the myocardium is from other cell types, including the tissue invading inflammatory cell component and cardiomyocytes [23-26].

As we do not detect reduced levels of TGFβ, or its receptor subtypes TGFβ-R1 and TGFβ-R2 gene expression, minimally detectable levels of secreted active or total TGFβ within the cells supernatant (<1 pg/mL) or reduced Smad 2 phosphorylation, we believe that stretch-mediated inhibition of TGFβ-induced cardiac fibroblast differentiation occurs independently of TGFβ receptor signaling or downstream of Smad 2. One alternative mechanism may involve ERK signaling as it has recently been reported that TGFβ mediated ERK phosphorylation under conditions of cyclical stretching can be impaired [27]. However, we do not observe reduced ERK phosphorylation when comparing the impact of short time point (15 and 45 min) TGFβ stimulation in cells that have been stretched for 72 h vs. non-stretched cells. Investigating the impact of chronic stimulation with TGFβ on ERK phosphorylation under mechanical stretch conditions would be of interest.

In parallel to stretch-induced attenuation of TGFβ response, we report an important novel observation that mechanical stretch can up-regulate the expression of both BNP and its receptor NPRA in human cardiac fibroblast cells. In addition, TGFβ stimulation in the presence of mechanical stretch can further enhance the observed BNP up-regulation. BNP has been reported to be synthesized in both animal and human cardiac fibroblasts [28-30], however the impact of stretch on its expression in these cells has not been noted to date. In addition, the impact of mechanical stretch on fibroblast expression of NPRA has not been explored. Although up-regulation of BNP expression following mechanical stretch of cardiomyocytes is well-documented, it is only more recently that mechanisms of this are being teased out. For example, a publication by Koivisto and colleagues highlight an important observation that ERK mediated activation of the muscle-CAT (MCAT) promoter element within the BNP gene contributes to stretch induced BNP up-regulation in cardiomyocytes [31]. Determining whether similar mechanisms occur in myofibroblasts would be important, however, MCAT activation within this cell type may be mediated by a different means [32].

These two observations that mechanical stretch can up-regulate BNP and NPRA in cardiac fibroblasts have important implications on our understanding of fibroblast biology in the context of the fibro-inhibitory effects of BNP [13,28]. Given that NPRA has recently been shown to play a protective role in an animal model of renal fibrosis [14] the relevance of stretch-regulated NPRA expression may extend to other organ systems. Although we do not show that endogenous production of BNP and enhanced NPRA expression directly accounts for attenuation of the TGFβ response, we do provide evidence that the anti-fibrotic effects of BNP are enhanced in stretched cells in line with the increase in NPRA expression. Western blot analysis of ASMA revealed that the observed inhibition of TGFβ-induced ASMA on stretched cardiac fibroblasts was further enhanced in the presence of exogenous recombinant BNP. Although exogenous BNP inhibits ASMA mRNA expression in static cardiac fibroblasts (data not shown) we demonstrate here that it has little effect on ASMA protein expression. Presumably this is due to the relatively low expression of NPRA on un-stretched cardiac fibroblasts. The impact of exogenous BNP on cardiac fibroblast function is highly relevant, given that the majority of BNP encountered by cardiac fibroblasts is likely to originate from the cardiomyocytes. However, fibroblast production of BNP may have an autocrine/paracrine effect and provide regulation at a local level. This could be particularly important in scar tissue, were fibroblast cells are isolated from neighboring myocytes within a sea of collagen.

Further work is required to delineate the precise mechanisms behind our findings, including the investigation of additional experimental time points, concentration of agonists used, and degree of mechanical stretch applied. Under normal physiological conditions, cardiac fibroblasts are exposed to cyclical mechanical stretch with each heart beat, at a frequency of approximately 1 Hz. Given the different cellular responses observed in this study when cells are exposed to cyclical mechanical stretch compared with static cells, additional studies expanding these observations in an enhanced pathological setting are warranted. In particular, it may be worth assessing the impact of increasing the degree of mechanical strain beyond 10% elongation or varying the level of TGFβ stimulation on the findings reported herein. Application of continuous cyclical mechanical stretch for durations beyond 72 h may provide a more accurate representation of hypertensive heart disease which is a chronic condition that exposes the myocardium to long periods of strain.

On this note, it must be highlighted that this study utilized one primary cell line from a healthy donor, and therefore to investigate these findings further one would need to take a more comprehensive approach using multiple primary cells lines from various donors spanning the age and cardiovascular health spectrum. It is possible that cardiac fibroblasts derived from a disease myocardium may behave differently, for example, the pathway controlling stretch up-regulation of BNP or NPRA may not be functional in diseased fibroblasts.

An additional benefit of using multiple primary cell lines from various donors would be to determine whether our observed findings are applicable to an array of phenotypically diverse fibroblast cells across the spectrum of myofibroblast differentiation. Extensive work carried out by Gabbiani and colleagues has highlighted an important intermediate phenotype between a fibroblast and a myofibroblast, termed the proto-myofibroblast [21,33]. The evolution of a fibroblast to a proto-myofibroblast is signified by the presence of beta and gamma actin stress fibers, with the development of cytoplasmic ASMA fibers only appearing in the fully differentiated myofibroblast [21,33]. The primary cardiac fibroblasts used in this study exhibited basal mRNA and protein expression of the contractile protein ASMA, however, ASMA stress fibers were not detected in control cells by immunocytochemistry (data not shown). It is therefore likely that these primary cells are on the continuum between a phenotypic fibroblast and a myofibroblast, and therefore may be more appropriately referred to as ‘proto-myofibroblasts’. With this in mind, the observed responses to mechanical stimuli herein may only be relevant to proto-myofibroblasts. This could also account for some of the conflicting findings with current literature as previously mentioned [19-21].

Cellular interpretation of a mechanical stimulus is likely to be influenced by the surrounding extracellular matrix, thus investigating the importance of integrin signaling during stretched cardiac fibroblast’s response to TGFβ and BNP should be considered [29,34,35]. For example, it is possible that the cellular responses we observed may be specific to cardiac fibroblasts interacting with extracellular matrix proteins containing RGD (Arg-Gly-Asp) domains, such as fibronectin and collagen IV [29,36]. Interestingly, NPRA has been demonstrated to interact with RGD-binding integrin receptors thereby enhancing BNP activation of cGMP in human cardiac fibroblasts [29,36].

## Conclusion

In summary, our data expand on the complex relationship between mechanical strain and cell/matrix interactions and human cardiac fibroblast physiology. We demonstrate that cell deformation via cyclical biaxial strain attenuates the effectiveness of TGFβ in inducing myofibroblast differentiation while simultaneously inducing BNP and NPRA production in human cardiac fibroblast cells. We confirm the inhibitory properties of BNP on TGFβ regulation of the myofibroblast differentiation marker ASMA and show that exogenous BNP treatment of stretched fibroblasts further reduces the potency of TGFβ. We postulate that the stretch-induced up-regulation of the natriuretic peptide system could account for these findings. These findings may describe a novel protective negative feedback mechanism for cardiac fibroblasts exposed to multiple pro-fibrotic stimuli and would encourage future work to directly link this observation, possibly through the use of siRNA experiments or NPRA receptor antagonists.

## Methods

### Cell culture

Primary human cardiac fibroblast cells from the adult ventricle (HCF) were purchased from ScienCell Research Laboratories. Primary cells were derived from a single female donor aged 20 years. Until required for experiments, cells were cultured and maintained in Dulbecco’s modified eagles medium (DMEM) (Gibco), supplemented with 10% fetal bovine serum (Gibco) and penicillin-streptomycin antibiotics (Gibco) in a 5% CO_2_ humidified incubator kept at 37 °C. All experiments involving cardiac fibroblasts were carried out under serum-free conditions.

### Transfection

HEK-293-T cells were used to generate a positive control for natriuretic peptide receptor A (NPRA; also known as guanylyl cyclase A, GCA) expression. Cells were purchased from American Type Culture Collection (ATCC) and were cultured in DMEM supplemented in the same manner as for HCF cells. HEK-293-T cells were transiently transfected in six well plates for 48 h with 1 μg/well of a pcDNA3_NPRA expression construct (kind gift from Dr Michaela Kuhn, University of Wurzburg, Germany) or empty vector using FuGENE (Roche) as recommended by the manufacturer’s instructions.

### Treatments

Where indicated, HCF cells were treated with 10 ng/mL human recombinant TGFβ1 (R&D Systems) for 72 h. For analysis of Smad phosphorylation, cells were treated with TGFβ1 for 15 or 45 min. Human recombinant BNP was purchased from American Peptide Company Inc. HCF cells were treated with recombinant BNP at a concentration of 1000nM for 72 h. BNP was added to the medium three times a day as previously described [13].

### Mechanical stretch

All mechanical stretch experiments were carried out on BioFlex six well culture plates (Dunn Labortechnik GmbH) coated with 2 μg/cm^2^ human fibronectin (Sigma). Cells were seeded and grown to a confluency of approximately 70% on the fibronectin-coated BioFlex culture plates prior to transfer onto the loading station of the FX-4000 T mechanical stretch machine (Flexcell International Corporation), and exposed to cyclic equibiaxial Heart Simulation strain (1 Hz, maximum elongation 10%) for 72 h. Control plates were exposed to the same conditions without being stretched.

### Quantitative real-time PCR (QPCR)

RNA isolation from cells was achieved using NucleoSpin RNA II Kit (Macherey-Nagel). First strand cDNA synthesis was carried out using SuperScript II RT (Invitrogen). QPCR primers were designed so that one of each primer pair was exon/exon boundary spanning to ensure only mature mRNA was amplified. The sequences of the gene-specific primers used are as follows; BNP, 5'-ACCGCAAAATGGTCCTCTAC-3′ (forward), 5′- CGCCTCAGCACTTTGCAG-3′ (reverse); NPR1, 5′-CGCAAAGGCCGAGTTATCTA-3′ (forward), 5′-AACGTAGTCCTCCCCACACA-3′ (reverse); ASMA, 5′-CGTTACTACTGCTGAGCGTGA-3′ (forward), 5′-AACGTTCATTTCCGATGGTG-3′ (reverse); collagen 1 α1 (COL1A1), 5′-GAACGCGTGTCATCCCTTGT-3′ (forward), 5′-GAACGAGGTAGTCTTTCAGCAACA-3′ (reverse); collagen 3 α1 (COL3A1), 5′- AACACGCAAGGCTGTGAGACT-3′ (forward), 5′- GAACGAGGTAGTCTTTCAGCAACA-3′ (reverse); TGFβ, 5′-CGACTCGCCAGAGTGGTTA-3′ (forward), 5′-GAACCCGTTGATGTCCACTT-3′ (reverse); TGFβ receptor type-1 (TGFβR1), 5′-ATTGCTGGACCAGTGTGCTT-3′ (forward), 5′-AAACCTGAGCCAGAACCTGA-3′ (reverse); TGFβ receptor type-2 (TGFβR2), 5′-AGTCGGATGTGGAAATGGAG-3′ (forward), 5′-GCTCATGCAGGATTTCTGGT-3′ (reverse). QPCR reactions were normalized by amplifying the same cDNA with GAPDH primers, 5′-ACAGTCAGCCGCATCTTCTT-3′ (forward), 5′-ACGACCAAATCCGTTGACTC-3′ (reverse).

QPCR was performed using Platinum SYBR Green qPCR SuperMix-UDG (Invitrogen). Amplification and detection were carried out using Mx3000P System (Stratagene). The PCR cycling program consisted of 40 three-step cycles of 15 s/95 °C, 30 s/T_A_, and 30 s/72 °C. Each sample was amplified in duplicate. In order to confirm signal specificity, a melting program was carried out after the PCR cycles were completed. The samples were quantified by comparison with a standard calibration curve created at the same time and the data was normalized by an internal control (GAPDH).

### Western blot analysis

Whole cell protein lysates were generated using RIPA Lysis Buffer (Millipore), containing a protease inhibitor cocktail (Roche). Protein concentrations were determined using the BCA Protein Assay Kit (Pierce). A total of 10–50 μg of whole cell lysates were denatured, reduced, and resolved on SDS-polyacrylamide gels by SDS-PAGE before transfer onto 0.45 μm pore size Immobilon-P polyvinylidene fluoride (PVDF) membranes (Millipore).

Membranes were incubated with blocking buffer (TBS, 0.25% Tween-20, 0.1% serum from species that secondary antibody was raised in, and 10% fat-free skimmed milk) for 1 h at room temperature. Membranes were subsequently probed overnight with either anti-NPRA (FabGennix Inc. International), anti-ASMA (Sigma), anti-phospho Smad2 (Cell Signaling Technologies), anti-total Smad2 (Cell Signaling Technologies), or anti-phospho ERK (Cell Signaling Technologies). Detection of the specific binding of the primary antibody was achieved using HRP-conjugated secondary antibodies, followed by signal detection with Immobilon Western chemiluminescent HRP substrate (Millipore) according to the manufacturer’s instructions. Anti-beta tubulin (Sigma) was used to verify equal loading.

### Immunoassay

Both total and active forms of TGFβ1 released by HCF were quantified in cell supernatants. An acid activation step was carried out for assessment of total TGFβ1. This involved incubating the sample in 1 N HCl for 10 min at room temperature, followed by neutralization with 1.2 N NaOH/0.5 M Hepes. This step was excluded when assaying for active TGFβ1 within the cell culture supernatant. Following sample preparation, TGFβ1 was quantified using the human active TGFβ1 ultra-sensitive immunoassay with electrochemiluminescence detection as instructed by the manufacturer (Meso Scale Discovery). This assay exhibits a large dynamic range suitable for quantifying pg/mL concentrations in the supernatants of the cultured cardiac fibroblasts.

### Immunofluorescence microscopy

HCF cells adhered to BioFlex wells were fixed in 4% paraformaldhyde for 20 min prior to being immuno-stained with either anti-NPRA (1:25) or immunoglobulin control at the same protein concentration, for 1 h at room temperature. Fluorescent detection of NPRA was achieved using Alexa Fluor 568 conjugated secondary antibody (Invitrogen), followed by nuclear staining with 4'-6-diamidino-2-phenylindole (DAPI). The silicone wells containing the immuno-stained cells were subsequently excised, mounted onto microscope slides, and visualized using fluorescent microscopy (Zeiss Axio Imager M1). Images were acquired at the same exposure time for comparison.

### Statistical analysis

Comparisons between the control and stretched groups were made using independent *t*-test or ANOVA (Tukey post-hoc analysis), where appropriate, with *P* values ≤0.05 considered statistically significant. All statistical calculations were performed using Graph Pad prism Software (Version 4, San Diego, CA, USA).

## Abbreviations

ASMA, Alpha-smooth muscle actin; BNP, B-type natriuretic peptide; ERK, Extracellular-signal-regulated kinases; HCF, Primary human adult cardiac fibroblast cells; NPRA, Natriuretic peptide receptor A; QPCR, Quantitative real-time PCR; TGFβ, Transforming growth factor beta; TGFβ-R, Transforming growth factor beta receptor.

## Competing interests

The authors declare that they have no competing interests.

## Authors’ contributions

CW, DP, MX, PC, and RN carried out the experiments and performed the data analysis. CW, DP, AS, ML, KM, and JB designed the experiments, interpreted the data, and wrote the final manuscript. All authors read and approved the final manuscript.
